# Proteinase-Activated Receptor 2 Is a Novel Regulator of TGF-β Signaling in Pancreatic Cancer

**DOI:** 10.3390/jcm5120111

**Published:** 2016-11-30

**Authors:** David Witte, Franziska Zeeh, Thomas Gädeken, Frank Gieseler, Bernhard H. Rauch, Utz Settmacher, Roland Kaufmann, Hendrik Lehnert, Hendrik Ungefroren

**Affiliations:** 1 First Department of Medicine, University Hospital Schleswig-Holstein (UKSH), and University of Lübeck, D-23538 Lübeck, Germany; davidwittemail@gmail.com (D.W.); franziskazeeh@gmx.de (F.Z.); T.Gaedeken@web.de (T.G.); frank.gieseler@uksh.de (F.G.); hendrik.lehnert@uni-luebeck.de (H.L.); 2 Department of General Pharmacology, Institute of Pharmacology, University Medicine Greifswald, D-17487 Greifswald, Germany; bernhard.rauch@uni-greifswald.de; 3 Department of General, Visceral and Vascular Surgery, Jena University Hospital, D-07747 Jena, Germany; utz.settmacher@med.uni-jena.de (U.S.); roland.kaufmann@med.uni-jena.de (R.K.)

**Keywords:** pancreatic carcinoma, signaling, TGF-β, ALK5, PAR2, myostatin, GDF15, cachexia

## Abstract

TGF-β has a dual role in tumorigenesis, acting as a tumor suppressor in normal cells and in the early stages of tumor development while promoting carcinogenesis and metastasis in advanced tumor stages. The final outcome of the TGF-β response is determined by cell-autonomous mechanisms and genetic alterations such as genomic instability and somatic mutations, but also by a plethora of external signals derived from the tumor microenvironment, such as cell-to-cell interactions, growth factors and extracellular matrix proteins and proteolytic enzymes. Serine proteinases mediate their cellular effects via activation of proteinase-activated receptors (PARs), a subclass of G protein-coupled receptors that are activated by proteolytic cleavage. We have recently identified PAR2 as a factor required for TGF-β1-dependent cell motility in ductal pancreatic adenocarcinoma (PDAC) cells. In this article, we review what is known on the TGF-β-PAR2 signaling crosstalk and its relevance for tumor growth and metastasis. Since PAR2 is activated through various serine proteinases, it may couple TGF-β signaling to a diverse range of other physiological processes, such as local inflammation, systemic coagulation or pathogen infection. Moreover, since PAR2 controls expression of the TGF-β type I receptor ALK5, PAR2 may also impact signaling by other TGF-β superfamily members that signal through ALK5, such as myostatin and GDF15/MIC-1. If so, PAR2 could represent a molecular linker between PDAC development and cancer-related cachexia.

## 1. Pancreatic Ductal Adenocarcinoma and TGF-β Signaling

Pancreatic ductal adenocarcinoma (PDAC) is one of the most fatal human cancers and its incidence is rising dramatically. Early diagnosis is hampered by the fact that symptoms are largely non-specific and PDAC therefore frequently presents at an advanced stage with extensive metastasis, which portends a poor prognosis. Unfortunately, only 15%–20% of patients have resectable tumors at diagnosis. Most patients have either a locally advanced or already metastasized tumor. The median survival is 8–12 months for patients with locally advanced disease and only 3–6 months for patients with metastases. The 5-year survival prognosis for patients with metastatic PDAC is about 8%—the lowest survival rate of all cancers and the fourth most common cause of cancer death [1]. Pancreatic cancer patients often suffer from venous thromboembolism [2] and cancer cachexia [3] which decreases life quality and enhances morbidity. Despite the introduction of new therapies, survival rates have not markedly improved in recent years. Metabolic risk factors or diseases associated with PDAC, amongst others, include chronic pancreatitis, obesity, and diabetes mellitus type II [1]. Biologically, PDAC is characterized by genomic instability and somatic mutations in *KRAS*, *p16^INK4A^*, *TP53*, and *DPC4*, together accounting for its aggressive nature. The *DPC4* encoded Smad4 protein is a central mediator of transforming growth factor (TGF)-β signaling and mutated in approximately 50% of invasive pancreatic carcinomas [4]. TGF-β signaling has a central role in the cancer progression of PDAC because (i) the TGF-β pathway naturally contains mutations and other well-defined alterations; and (ii) it belongs to only four signaling pathways that are mutated (with at least one gene) in 100% of tumors [5]. Moreover, mouse models of PDAC have shown that mutations in the pathway e.g., in Smad4 and TGF-β type II receptor (TβRII) are causative in the development of aggressive/metastatic PDAC by cooperating with members of the Ras/Rac family of small GTPases and other non-Smad pathways to induce neoangiogenesis, host immune suppression, invasion and metastasis [6]. As can be expected from its diverse functions in normal cells and in cancer cells, TGF-β signaling is controlled in a complex fashion and by a plethora of both positive and negative factors. Their delicate balance in expression or activity allows the cell to fine-tune activation of the pathway according to biological needs and to protect itself from overactivation which may result in cellular stress and loss of homeostasis.

## 2. PAR2 and TGF-β Signaling Have Similar Functions: Circumstantial Evidence for a Functional Interaction

Proteinase-activated receptor 2 (PAR2) is a prototype member of a subfamily of G protein-coupled receptors, the proteinase-activated receptors (PARs) that control a number of (patho)physiological processes, such as vasoregulation, nociception, inflammation, and tissue regeneration [7,8,9]. It is highly expressed in the pancreas, is a key regulator of pancreatic exocrine secretion [10], is upregulated in a rat model of chronic pancreatitis [11] and is involved in liver fibrosis [12]. This is interesting because chronic pancreatitis is considered a risk factor for PDAC [1] while fibrosis (desmoplasia) is a hallmark of PDAC (see below). PARs are characterized by a specific activation mechanism, involving serine proteinase-mediated cleavage in a specific domain of the extracellular N-terminus and exposure of a “tethered ligand” that binds to the receptor and activates it [7,8]. Once activated, PAR2 binds to G_αq_, G_αi_ and G_α12/13_ subtypes and induces activation of ERK1/2, mobilization of intracellular Ca^2+^, RhoGEF-mediated Rho and Rac signals [7] but can also signal independently of G proteins via the multifunctional adapter protein β-arrestin-2 [7,9]. Besides trypsin released from the pancreatic acinar cells during inflammatory tissue damage, PAR2 is activated by tissue factor/factor VII/factor Xa complexes that may thus be present along with trypsin, and TGF-β in the tumor microenvironment. This is relevant since diagnosis of PDAC, especially in advanced stages, is often associated with a particularly high rate of venous thromboembolism [13] which occurs in over one-third of contemporary pancreatic cancer patients and, whether symptomatic or incidental, is strongly associated with worsened mortality [14].

Interestingly, PAR2 and TGF-β signaling share several functions in common, e.g., a profibrotic role in the liver [12], the ability to stimulate pro-fibrogenic cytokines, to induce the proliferation and differentiation of fibroblasts into pancreatic myofibroblasts and to stimulate production of matrix proteins in human hepatic stellate cells (HSCs) in vitro and in vivo [12]. Moreover, PAR2 and TGF-β/ALK5 activate the same intracellular signaling pathways (ERK1/2 and p38 mitogen-activated protein kinase (MAPK), protein kinase C, Rac/PAK, c-Src, NFκB) and intermediates (reactive oxygen species, Ca^2+^) [7], while others are receptor (class)-specific such as phosphatidylinositol (3–5)-trisphosphate and β-arrestin-2 signaling for PAR2 [7,9] and signaling through Smad proteins for TGF-β/ALK5 [4]. Interestingly, PAR2 and TGF-β1 mutually regulate their expression with PAR2 increasing the synthesis of TGF-β1 in HSCs [12] and TGF-β1, in turn, inducing PAR2 expression in endometric stromal cells [15].

With respect to cancer, both TGF-β and PAR2 have been associated with cancer development and progression, e.g., tumor growth, migration, invasion and metastasis in various tumor entities [16,17,18] including PDAC [19,20]. With respect to primary tumor growth, PAR2^−/−^ mice subcutaneously inoculated with B16 melanoma cells exhibited larger primary tumors, while orthotopic xenografts of the human PDAC cell line Panc1 expressing a kinase-active mutant of the TGF-β type I receptor ALK5 in *scid*/*bg* mice presented with *smaller* primary tumors. However, in PAR2^−/−^ mice, the transplanted B16 melanoma cells generated *less* distant metastases and allowed for a longer survival time of their hosts compared with strain-matched wild-type controls [21], while Panc1 xenografts with kinase-active ALK5 generated *more* distant metastases [22]. Although derived from two different animal models and differing in the PAR2 expressing compartment (stromal vs. tumor cell), the data nevertheless suggest that both TGF-β/ALK5 and PAR2 *inhibited* primary tumor growth but *promoted* metastatic dissemination. The clinical relevance of PAR2 expression is particularly evident in gastric cancer, in which PAR2 expression correlated with the depth of wall, lymphatic, and venous invasion as well as liver metastasis. Not surprisingly, patients with PAR2-positive tumors had a significantly poorer prognosis than those with expression-negative tumors [23].

PDAC tissue is characterized by pronounced desmoplasia, e.g., a high amount of extracellular matrix intermingled with host stromal cells, such as pancreatic myofibroblasts, cancer-associated fibroblasts, smooth muscle cells and various types of immune cells. The desmoplastic/fibrotic reaction is caused, to a large extent, by TGF-β but PAR2 may also contribute due to its known profibrotic role and the high amount of serine proteinases secreted by both cancer and stromal cells. Moreover, PDAC-derived cells display a particularly high expression of both PAR2 and TGF-β1 which can mutually control their expression [12,15] and in advanced cancer stages TGF-β can auto-induce its expression. This eventually results in the accumulation of TGF-β in the tumor tissue through a positive feedback loop and eventually escalation of angiogenesis, epithelial-mesenchymal transition (EMT), migration, invasion and metastasis formation [24]. TGF-β and various PAR2-activating serine proteinases are important constituents of the tumor microenvironment where they are involved in the dialogue between cancer cells and neighboring stromal cells [25,26]. The colocalization, co- and mutual regulation, and broad spectrum in similar (patho)physiological functions on the one hand and the observation that PAR2 functionally interacts with members of other receptor families, particularly tyrosine kinase receptors (reviewed in [8]) on the other hand pointed to a more intimate and functional interaction between TGF-β and PAR2 signaling.

**Figure 1:**
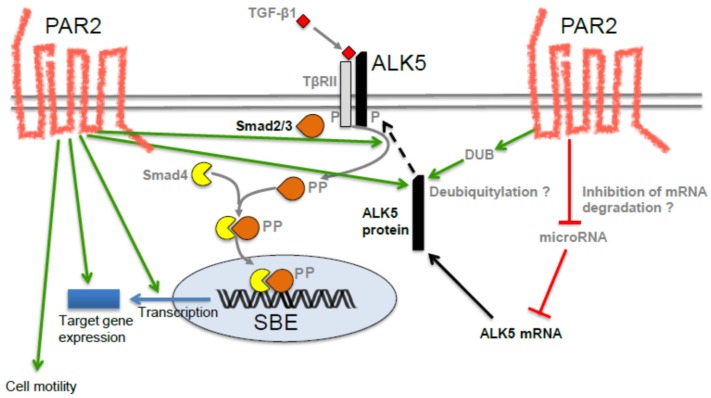
Cartoon showing potential mechanisms of the PAR2-TGF-β/ALK5 interaction. In the left part of the figure, the various effects of PAR2 on TGF-β signaling are indicated by green arrows such as a rise in ALK5 protein, R-Smad phosphorylation, Smad-mediated transcription, target gene expression and cell motility. In the right part, possible but as yet unproven mechanisms are shown that may cause an increase in ALK5 abundance, such as deubiquitylation (eventually resulting in ALK5 protein half-life extension) or inhibition of an ALK5-targeting microRNA. Stimulatory interactions are indicated by green arrows and inhibitory interactions by red lines. Anterograde transport of ALK5 protein to the cell surface is marked by a black stippled arrow. P, phosphate residue; SBE, Smad binding element; DUB, deubiquitylating enzyme.

More direct evidence for this came from a study showing that peptide agonist-mediated activation of PAR2 in human kidney epithelial cells induced the expression of connective tissue growth factor (CTGF) in vitro and that this induction was dependent on ALK5 [27]. Prompted by these observations, we pursued the possibility that signaling by TGF-β1 is, in turn, dependent on PAR2. Using siRNA-mediated silencing of PAR2 and real-time analysis of random cell migration and invasion, we have verified that PAR2 protein expression was required for TGF-β1-mediated cell motility in several (TGF-β-sensitive) cell lines of PDAC origin [28]. In search of the molecular basis, we found that PAR2 depletion blunted the TGF-β response of several TGF-β target genes involved in the regulation of cell motility, including the prototypical TGF-β response gene *serpine1*, encoding plasminogen activator inhibitor-1 (PAI-1) [28]. Promoter reporter gene assays with TGF-β-responsive plasmids (p3TP-Lux, p6SBE-Luc, p(CAGA)_12_-Luc) in Panc1, Colo357 and HEK293T cells showed that after siRNA-mediated silencing of PAR2, the TGF-β -mediated transcriptional activity of all three reporters was either completely lost or severely reduced, suggesting that PAR2 is required for general transcriptional activity induced by TGF-β1 [28]. Since the reporter plasmids were all Smad-responsive, we wanted to know if PAR2 depletion was necessary for activation of receptor-regulated Smads, Smad2 and Smad3. Interestingly, PAR2-depleted Panc1, Colo357, and IMIM-PC1 cells were no longer able to respond to TGF-β1 with phosphorylation of the C-terminal Ser-Ser-X-Ser (SSXS) motif in Smad3 and Smad2 [28]. Likewise, PAR2-depleted cells failed to phosphorylate p38 MAPK in response to TGF-β1 stimulation [28]. Together, these findings strongly suggest that a functional cooperation between the TGF-β receptor(s) and PAR2 is required for activation of Smad and non-Smad signaling. Since Smad2 and Smad3 are direct substrates of ALK5 which is in turn phosphorylated by TβRII, we reasoned that PAR2 could affect the expression of activity of either ALK5 or TβRII. When we monitored the abundance of both receptors at the protein level, we noted a dramatically lower expression of ALK5 but not TβRII in PAR2-depleted cells, indicating that PAR2 specifically targets ALK5. Moreover, reconstitution by ectopic expression of wild-type and kinase-active versions of ALK5 (ALK5-T204D) but not of a Smad binding-defective mutant (RImL45-T204D) was able to rescue cells from losing sensitivity to TGF-β1 as demonstrated by their ability to restore TGF-β1-induced Smad3C phosphorylation, reporter gene activity and cell migration [28]. This proved that PAR2 promotes TGF-β signaling by sustaining protein expression of ALK5 which may occur at the mRNA level, either through an increase in de novo transcription of *TGFBRI* or via suppression of a microRNA that targets ALK5 mRNA for degradation. Alternatively, deubiquitylation by deubiquitylating enzymes may stabilize ALK5 protein through inhibition of proteosomal degradation [28]. The current knowledge on the mechanistic interaction between PAR2 and TGF-β signaling has been summarized in Figure 1 [28].

## 3. Possible Role of PAR2 in TGF-β-Induced EMT and EMT-Associated Alterations

EMT is considered a prerequisite for cells to become motile and invasive. Given the crucial role of PAR2 in the TGF-β control of cell motility, it is conceivable that PAR2 also impacts TGF-β-induced EMT as well as EMT-associated changes, such as adoption of a spindle-shaped morphology, proliferation arrest, autoinduction of TGF-β1 ligand, chemoresistance and cancer stem cell (CSC) formation. With respect to EMT-associated gene expression, we observed that siRNA-mediated depletion of PAR2 in Panc1 cells resulted in reduced sensitivity to TGF-β1 stimulation of several EMT-associated genes and stem cell markers [29]. Interestingly, cells depleted of PAR2 protein lost the ability to autoinduce TGF-β1 at both the mRNA and protein level in response to stimulation with exogenous TGF-β, suggesting that inhibiting PAR2 expression or function is a suitable strategy to interfere with a TGF-β autostimulatory loop in the tumor tissue. In a mouse model of PDAC, it has been shown that the EMT program induced a decrease in proliferation [30]. Regarding a possible role in TGF-β-induced EMT, it was exciting to see that TGF-β1-induced growth inhibition was abolished in PAR2-depleted Panc1 and Colo357 cells. Notably, the failure of TGF-β1 to suppress cell growth in PAR2-deficient cells correlated with its failure to upregulate the cyclin-dependent kinase inhibitor p21^WAF1^ [31].

TGF-β-induced EMT is associated with resistance to radiotherapy and treatment with chemotherapeutic drugs, such as gemcitabine that targets mitotically active cells. Resistance to gemcitabine in PDAC patients and in the orthotopic mouse models of PDAC ([30,32] and references therein) is due to reduced proliferation and reduced apoptosis sensitivity. Since PAR2-depleted PDAC cells are expected to exhibit greater mitotic activity due to the loss of TGF-β-induced growth arrest, inhibiting PAR2 expression and/or function may restore the cell’s sensitivity to gemcitabine chemotherapy. Furthermore, we recently discovered that TGF-β1 downregulates the type I receptor for TRAIL (TNF receptor apoptosis-inducing ligand), TRAIL-R1/DR4, in a Smad4-dependent fashion [33]. TRAIL-R1 is utilized by TRAIL to selectively induce apoptosis in cancer cells [33]. Preliminary data indicate that TRAIL-R1 downregulation by TGF-β1 is lost upon cellular PAR2 depletion. If this results in reduced sensitivity of PDAC-derived cells to TRAIL-induced apoptosis, then the PAR2-TGF-β interaction would be crucially involved in a novel tumor-promoting function of TGF-β—tumor escape from immune surveillance and resistance to TRAIL- or TRAIL-R1-based therapy regimens for treatment of PDAC [33].

Activation of TGF-β is an early and persistent event in tumor response to radiation and TGF-β signaling controls an effective DNA damage response (DDR). TGF-β depletion compromises cell survival in response to radiation and impairs activation of the DDR because of severely reduced activity of ataxia telangiectasia mutated (ATM), a serine/threonine protein kinase that is rapidly activated by DNA double-strand breaks. The crosstalk between the TGF-β and ATM pathways in the DDR is dependent on Smad2, Smad7 and TGF-β receptor type I/ALK5 in human fibroblasts and epithelial cells. Thus, TGF-β pathway inhibition has therapeutic implications for improving the response to DNA damage-inducing therapy [34,35]. We and others have recently demonstrated that ionizing radiation induced in lung adenocarcinoma and PDAC-derived cells in vitro TGF-β secretion and EMT-associated alterations such as growth arrest and a dramatically increased migration potential [36]. The EMT program may account for enhanced resistance to further irradiation episodes or chemoresistance in case of combined radio- and chemotherapy regimens [32]. In the worst case, EMT induction eventually results in radiation-induced fibrosis [37] or even in the radiotherapy-associated formation of secondary malignancies. Given this important role of TGF-β/ALK5/Smad signaling in the DDR on the one hand and regulation of TGF-β/ALK5/Smads by PAR2 on the other hand, we would like to postulate a crucial role of PAR2 in DDR regulation by TGF-β. In addition, we are currently analyzing if depletion of PAR2 by either siRNA transfection or CRISPR/Cas9 technology can sensitize tumor cells to apoptotic cell death following irradiation and can mimic conventional forms of TGF-β pathway inhibition, e.g., ALK5 kinase inhibition with small molecules.

## 4. The PAR2-TGF-β Crosstalk Also Operates in Non-Malignant Cells of Human and Murine Origin

So far, we have analyzed the role of PAR2 in PDAC-derived cells which stand out by particularly high endogenous expression of both PAR2 and TGF-β ligand, yet data on the TGF-β -PAR2 signaling crosstalk in non-malignant cells were not available. In the course of a study analyzing the role of host cell-derived PAR2 in hepatocellular carcinoma progression, we utilized the (spontaneously immortalized) hepatic stellate cell line LX2, stably transfected with a PAR2 shRNA. Intriguingly, these cells were unable to respond to TGF-β1 stimulation with phosphorylation of the C-terminal SSXS motif in Smad3 [38]. Likewise, the immortalized, non-tumorigenic keratinocyte cell line HaCaT transiently transfected with PAR2 siRNA was unable to activate Smad2/3 and cell migration and to sustain readily detectable levels of ALK5 protein after TGF-β1 challenge [28]. Finally, we employed freshly isolated murine aortic smooth muscle cells (ASMCs) from wild-type and PAR2^−/−^ mice in ex vivo cultures to see whether the TGF-β-PAR2 interaction was also operating in normal cells of non-human origin. Notably, the TGF-β responsiveness of *serpine1* encoding plasminogen activator-inhibitor type 1 (PAI-1) was greatly reduced in ASMCs from PAR2^−/−^ mice when compared to that of wild-type mice. Together, these data indicate that the TGF-β-PAR2 signaling crosstalk is not restricted to malignant (tumor) cells and to human cells, and is likely not a cell culture artifact since it is also observed in short-term cultures of smooth muscle cells ex vivo.

## 5. Potential Therapeutic Implications of the TGF-β-PAR2 Interaction

Prompted by the essential role of PAR2 for TGF-β signaling, we would like to consider some scenarios in which disruption of the PAR2-TGF-β crosstalk could translate into future treatment strategies for PDAC. Given the potent proinvasive and prometastatic effects of TGF-β and PAR2, inhibiting PAR2 expression or function in the tumoral or stromal compartments of PDAC tumors is likely to have a strong impact on metastatic disease. This comes on the one hand from inhibition of PAR2-driven (independent of TGF-β) invasion in response to activation by serine proteinases including kallekrein-related peptidases and blood coagulation enzymes [39] of the tumor microenvironment and on the other hand from disruption of the PAR2-TGF-β crosstalk. Moreover, both PAR2 [40] and TGF-β [41] are able to promote angiogenesis through VEGF expression and release and are thus essential for tumor survival under hypoxic conditions of the microenvironment. PAR2 also maintains a constitutive high level of HIF-1α for angiogenesis promotion and this also explains the high propensity for metastatic dissemination of pancreatic cancer cells in hypoxic regions [40]. Previous results have also shown that PAR2 depletion abrogated the ability of TGF-β1 to induce the expression of extracellular matrix genes in vitro [28]. If inhibitors of PAR2 are able to halt this profibrotic TGF-β effect in vivo, then they would hold great promise in fighting desmoplasia, one of the hallmarks of PDAC.

As mentioned above, several standard therapies that are based on eradicating mitotically active cells may be promoted by disruption of the TGF-β-PAR2 interaction. For instance, TGF-β pathway inhibition has therapeutic implications for improving the response to DNA damage-inducing therapy [34,35]. In addition to using classical TGF-β signaling inhibitors [42] sensitization of tumor cells to either cell cycle arrest or apoptosis following irradiation may be accomplished by inhibition of PAR2 expression or function. Enhanced proliferation of the tumor cells that eventually results from the loss of TGF-β-induced growth arrest in PAR2-depleted/inhibited cells may also increase the cell’s sensitivity to gemcitabine chemotherapy. Moreover, it is conceivable that potential anti-apoptotic functions of TGF-β, such as downregulation of TRAIL-R1, are blunted by PAR2 inhibition and lead to improved success of TRAIL- or TRAIL-R1-based therapy regimens or a combination of TRAIL agonist with gemcitabine [40].

Although their development proved challenging, several small-molecule PAR2 antagonists are now available that were based on the tethered ligand motif of PAR2 and block receptor activation caused by the prototype ligand trypsin [43], e.g., two peptides and the compounds, ENMD-1068, K-14585, and GB88. ENMD-1068 attenuates PAR2-mediated murine joint inflammation in vivo, while K-14585 and GB88 inhibit PAR2-dependent calcium and pro-inflammatory signaling and effectively attenuate inflammation in a rat model of colitis, respectively. K-14585 and GB88 are both signal pathway-specific antagonists that inhibit PAR2-induced intracellular calcium release, cyclic adenosine monophosphate stimulation, receptor internalization and pro-inflammatory cytokine release without affecting PAR2-mediated MAPK phosphorylation. Other PAR2 antagonists are likely to share this property of ‘biased’ antagonism [43].

These newly developed PAR2 antagonists may be applied alone or in combination with TGF-β signaling inhibitors that are either in clinical use or are evaluated in clinical trials for their anti-tumor activity [42]. The combination is expected to be more effective in suppressing tumor progression by disrupting neoangiogenesis, desmoplasia, and metastasis in advanced PDAC tumors. Mechanistically, this is achieved by blockage of TGF-β-dependent responses but also by disrupting mutual regulation and auto-induction of TGF-β and PAR2 expression [12,15]. Moreover, it has recently been shown that PAR2 agonist-dependent CTGF expression in renal cells is ALK5-dependent [27]. If ALK5 transactivation of PAR2 also operates in vivo in the tumor tissue to drive PAR2-dependent invasion and metastasis, then small molecule inhibitors of the ALK5 kinase, such as SB431542, LY2157299, or SD-208 would add another level of potency to a combined TGF-β/PAR2 inhibition approach. Given that death from cancer results from metastatic disease rather than the primary tumor, patients may greatly benefit from these combined therapies.

## 6. Possible Involvement of PAR2 in Signal Transduction by Other TGF-β Family Members: Implications for a Role of PAR2 in Cancer-Related Cachexia

Our studies have shown that PAR2 in various types of malignant and non-malignant cells facilitates TGF-β1 signaling by sustaining the expression of ALK5. However, the PAR2 effect must not be specific to signaling by TGF-βs. Rather, PAR2 may enhance signaling by other members from the TGF-β superfamily that utilize ALK5 and Smad2/Smad3, namely myostatin [44] and GDF15/MIC-1 [45,46].

Myostatin is an extracellular cytokine, mostly expressed in skeletal muscles and known to play a crucial role in the negative regulation of muscle mass. Upon the binding to activin type IIB receptor and ALK5 (or ALK4), myostatin can induce phosphorylation of Smad2/Smad3 and several other signaling cascades resulting in the upregulation of the atrogenes and downregulation of genes important for myogenesis [47]. GDF15/MIC-1 is widely distributed in mammalian tissues and has multiple functions in various pathologies including inflammation, cancer, and obesity [40]. Intriguingly, both myostatin and GDF15 have been associated with cachexia, a common and often fatal condition which occurs most commonly in advanced cancer. Myostatin upregulation was observed in the pathogenesis of muscle wasting during cancer-associated cachexia [47]. GDF15 is produced in large amounts by cancer cells and may act on feeding centres in the hypothalamus and brainstem to cause anorexia and eventually cachexia leading to loss of lean and fat mass [45].

Since both PAR2 and ALK5 are expressed by skeletal muscle [48], it is conceivable that serine proteinases released by the tumor tissue (either malignant epithelial cells, stromal cells or immune cells) activate PAR2 and further enhance myostatin signaling in muscle cells, ultimately leading to muscle wasting. Moreover, since PAR2 can be activated by FXa, venous thromboembolism via PAR2 activation may directly enhance myostatin and/or GDF15 signaling and cachexia. A similar scenario is conceivable for serine proteinases released during inflammatory diseases that precede cancer development, such as chronic pancreatitis or obesity, or proteinases released by pathogens, such as viruses and bacteria [49].

## 7. Conclusions

We have identified PAR2 as an essential component of TGF-β signaling in both malignant and non-malignant cells. By sustaining expression of the TGF-β type I receptor ALK5, PAR2 determines activity of the canonical Smad pathway and those non-Smad pathways that are initiated via ALK5. Moreover, the ability of PAR2 to control ALK5 protein abundance suggests the possibility that PAR2 can enhance the cellular actions of other TGF-β superfamily members, such as myostatin and GDF15, eventually leading to cancer-cachexia and muscle wasting. Since PAR2 is activated through various serine proteinases, it may couple TGF-β superfamily signaling in a direct fashion to a diverse range of other physiological processes, such as local inflammation, systemic coagulation or the innate immune response to viral or bacterial infection.
